# Xiaoyao Pill alleviates ulcerative colitis by inhibiting ferroptosis of enterocytes via activating Nrf2/Gpx4 signaling pathway

**DOI:** 10.3389/fphar.2025.1702452

**Published:** 2025-12-04

**Authors:** Zehua Zhou, Xueting Xing, Ruomei Zhang, Xiaoqing Zhang

**Affiliations:** 1 The International Peace Maternity and Child Health Hospital, School of Medicine, Shanghai Jiao Tong University, Shanghai, China; 2 Shanghai Key Laboratory of Embryo Original Diseases, Shanghai, China

**Keywords:** Xiaoyao pill, ulcerative colitis, ferroptosis, Nrf2/GPX4 signaling pathway, oxidativestress

## Abstract

**Background:**

The treatment of ulcerative colitis (UC) remains a huge challenge worldwide. Xiaoyao Pill (XYP) is a classic TCM formula, which possesses several benefits, including soothing the liver and invigorating the spleen. However, its protective effect on UC and its underlying mechanisms are unknown. Purpose: Here we explored the protective effect and underlying mechanism of XYP against dextran sulfate sodium (DSS)-induced colitis in mice.

**Methods:**

The experimental colitis was established by adding 3% DSS on drinking water of mice and the effects of XYP (0.32 and 0.64 mg/kg/d, i.g., by 10 days) in colon tissues was analyzed. Transcriptomic analysis elucidated therapeutic targets, subsequently validated through molecular techniques and cellular assays. NCM460 cell was induced by RSL3 to detect the effect of XYP on ferroptosis and the underlying mechanism. Pathological damage was determined by H&E. Indicators related to intestinal permeability were detected by immunohistochemistry and immunofluorescence. Cytokines levels (TNF-α、IL-1β and IL-6), antioxidant enzymes activities (MDA, SOD and GSH) from colon tissues of each group mice, the level of Fe^2+^ Cytokines levels and Gpx4 activity from colon tissues of each group mice or cells were detected by ELISA. Intracellular ROS levels in each group of cells were detected by H_2_DCFDA fluorescence staining. Finally, the key mediating role of nuclear factor erythroid 2-like 2 (Nrf2) in the XYP treatment was explored by cell transfection using siRNA or plasmid injection.

**Results:**

The results indicated that XYP significantly attenuated DSS-induced colon pathological damage, intestinal barrier, cytokines levels, and increased the antioxidant enzymes activities. Transcriptomic analyses illustrated that XYP might alleviate UC by inhibiting ferroptosis. Moreover, XYP attenuated ferroptosis in DSS-induced colon injury and regulated Nrf2/Gpx4 signaling pathway in DSS-induced mice. Mechanistic experiments verified that XYP activated Nrf2 *in vitro*.

**Conclusion:**

Taken together, this study evaluates that XYP alleviates DSS-induced colitis mice by inhibiting ferroptosis of enterocytes and its protective effects are associated with activating the Nrf2/Gpx4 signaling pathway.

## 1 Introduction

Ulcerative colitis (UC) is an inflammatory bowel disease (IBD) characterized by chronic inflammation of the gastrointestinal tract, primarily affecting the colon and rectum (Le Berre et al., 2020). It is clinically characterized by abdominal pain, constant, and persistent diarrhea, which is a recurrent and chronic inflammatory disease. Systemic therapies, including anti-tumor necrosis factor alpha agents, immunosuppressants, and corticosteroids are usually used to treat UC, however, it is not very efficacious for patients. Furthermore, these may be raised the risk for infections, and other side effects undesirably (Kaplan, 2015). Recently, aminosalicylates is the most common drug for UC treatment, which is also not ideal drug and exist side effects (Sandborn et al., 2020). Thus, there is a clear need for the development of alternative candidates that restrict these medication hangovers of systemic immunosuppression and exhibit long-term competence in UC patients.

Xiaoyao Pill (XYP) is a classic TCM formula that was first recorded in “*Prescriptions of Peaceful Benevolent Dispensary*” during the Chinese Song dynasty (960–1127 CE). XYP reportedly provides several benefits, such as soothing the liver, invigorating the spleen, and promoting blood circulation. XYP has been used for hundreds of years to treat mental disorders, such as depression and anxiety. Recent studies have documented the beneficial effects of XYP in animal models of several diseases (Fan et al., 2024; Zhang ZW. et al., 2023; Fang et al., 2020). There is evidence shown that XYP may regulate the brain-gut axis function and improve intestinal and depressive symptoms in IBD model mice (Yu et al., 2025). The mechanism may be through inhibiting the expression of proteins related to the intestinal ACT1/TRAF6/P38MAPK/AP-1 signaling pathway while activating the brain’s DRD2/TH signaling pathway and increasing dopamine release in the brain. In addition, recent reviews have assessed the clinical evidence supporting the efficacy and safety of XYP for functional dyspepsia (Ha et al., 2023). However, the protective effect of XYP in treating UC remains unclear. The specific mechanisms underlying the therapeutic effects are also needed to be explored.

Currently, research on TCM formulas often lacks clear targets and mechanisms of action. RNA sequencing (RNA-seq), a newly developed technology, provided a more complex and unprejudiced perspective of the dynamic and complicated nature of the transcriptome by obtaining gene information by high-throughput sequencing. Additionally, RNA-seq can recognize a broader spectrum of expression levels with reduced samples, which has been throughout utilized in the biological activity’s research (Yao et al., 2025). This approach provides genome-wide gene expression information, facilitating the capture of drug effects on multiple genes, pathways, and biological processes, by integrating pharmacological experiments, the study enhances prediction accuracy and feasibility (Wei et al., 2025).

**FIGURE 1:**
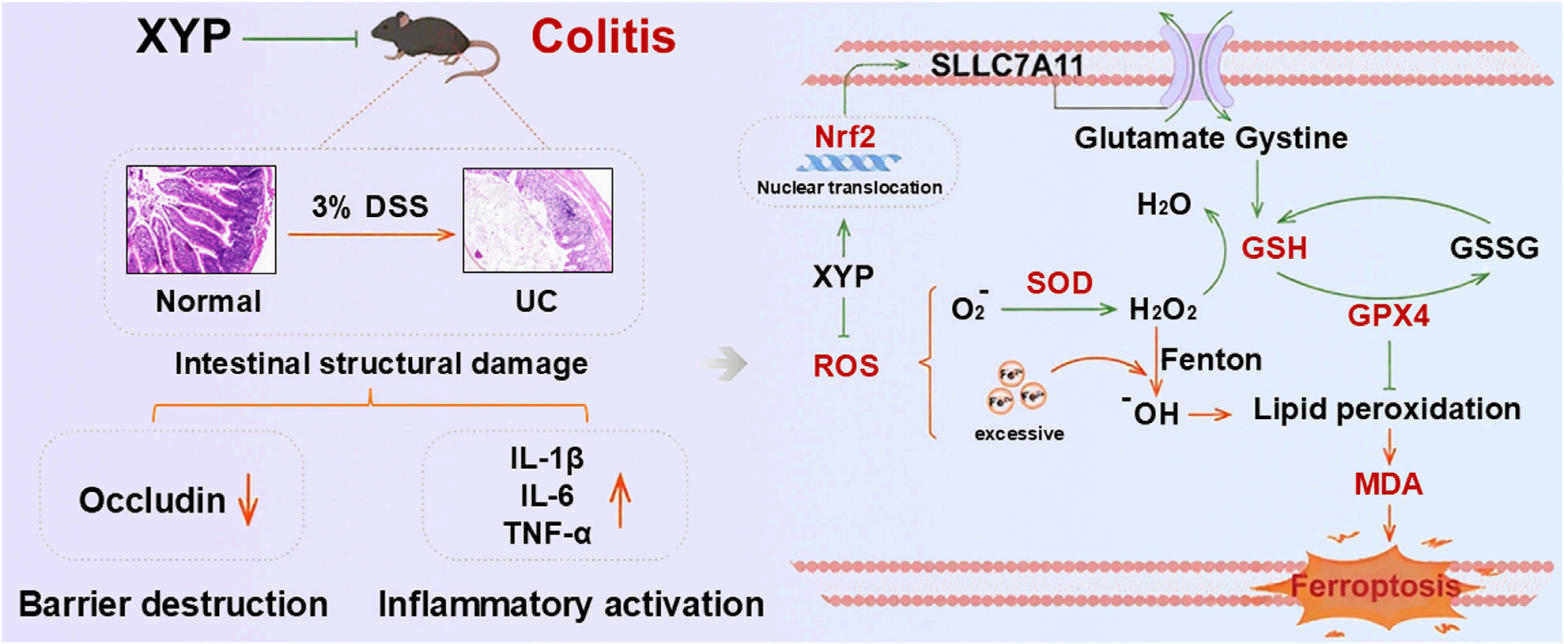
The mechanism diagram of XYP against ferroptosis in UC.

Therefore, this study aims to evaluate the efficacy of XYP in treating UC. Subsequently, transcriptomic analysis was conducted on the intestines of UC model mice intervened by XYP, speculating on its potential mechanism of action. Pharmacological experiments are conducted to further confirmed the result of transcriptomic analysis. The workflow is shown in Figure 1.

## 2 Materials and methods

### 2.1 Materials, reagents and chemicals

XYP were purchased from Shanghai Wanshicheng Pharmaceutical Co.,Ltd (batch no: 2025060310, National Drug Approval Number: Z20013180). The composition of XYPs was showed in the Pharmacopoeia of the People’s Republic of China (Xu et al., 2021) including the root of *Bupleurum chinense* DC., the root of *Angelica sinensis* (Oliv.) iels, the root of *Paeonia lactiflora* Pall., the sclerotia of *Poria cocos* (Schw.) Wolf (a kind of fungus), the rhizome of *Glycyrrhiza uralensis* Fisch., the leaves of *Mentha haplocalyx* Briq., and the rhizome of *Atractylis lancea* var. *chinensis* (Bunge) Kitam., in a ratio of 5:5:5:5:4:1:5. The method of extraction was showed in the Pharmacopoeia of the People’s Republic of China (Xu et al., 2021). Mesalazine (Mes) was bought from Shanghai Ethypharm Pharmaceutical Co.,Ltd. Dextran sulfate sodium (DSS, MW; 36–50 kDa) was purchased from Chem-Lab (Zedelgem, Belgium). Ferrostatin-1 (Fer-1) was purchased from Bide Pharmatech Ltd. (Shanghai, China). RSL3 was from Sigma-Aldrich (St. Louis, MO, USA). All antibodies and their details used in Western blotting analysis are presented in Table 1.

**TABLE 1:** Antibody Information Used in Western blotting analysis.

Antibody	Dilution	Manufacturer	Cat. no
Rabbit occludin polyclonal antibody	1:10,000	Proteintech	27260-1-AP
Rabbit Nrf2 monoclonal antibody	1:1 000	Abcam	ab313825
Rabbit COX2 monoclonal antibody	1:3 000	Abcam	ab179800
Rabbit Keap1 monoclonal antibody	1:1 000	Abcam	ab119403
Rabbit GPX4 polyclonal antibody	1:1 000	Proteintech	30388-1-AP
Rabbit GAPDH polyclonal antibody	1:10,000	Proteintech	20536-1-AP
Rabbit histone H3 polyclonal antibody	1:1 000	Abcam	ab308373
HRP conjugated goat anti-rabbit IgG (H + L)	1:10,000	Servicebio	GB23303

### 2.2 XYP quality control (QC)

The analysis was performed by High-Performance Liquid Chromatography (HPLC) (Agilent, US). The column was C18 column (4.6 × 250 mm, 5 μm), and the chromatographic separation conditions were as follows: column temperature: 25 °C; flowrate: 1.0 mL/min; mobile phase: acetonitrile+0.1% phosphoric acid (15 : 85); stock solutions of XYP were prepared by dissolving 0.4 g of analyte in 25 mL dilute ethanol (Fang et al., 2020). The content in XYP was determined by quantitation paeoniflorin (C_23_H_28_O_11_). Paeoniflorin content should not be less than 4.0 mg in 1.0 g of concentrated pills according to the Pharmacopoeia of the People’s Republic of China 2020 Edition. The content of paeoniflorin in 1.0 g XYP is 6.5 mg (Supplementary File 1).

### 2.3 Experimental animals

Male C57BL/6 mice (8 weeks, 20 ± 2 g) were obtained from Shanghai Laboratory Animal Co., Ltd. (production license: PZSHUTCM2302210003). Mice were housed in the Experimental Animal Center of Shanghai University of Traditional Chinese Medicine, receiving standard chow and water, and maintained under controlled conditions (22 °C ± 2 °C, 12 h light-dark cycle). After a 3 days acclimatization period, mice were matched by body weight.

### 2.4 Modeling and treatment methods for UC model

**FIGURE 2:**
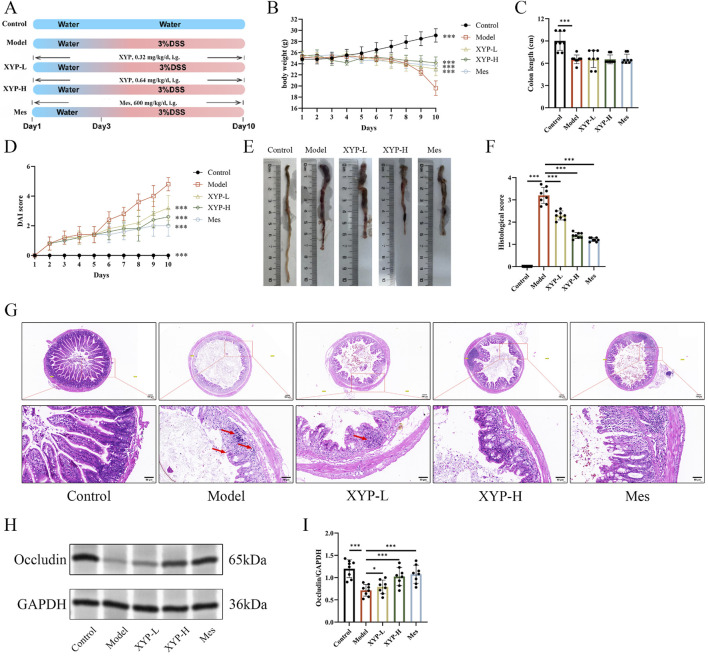
Amelioration of XYP on UC mice. **(A)** Experimental diagram of the establishment of UC model and drug treatment. **(B)** Changes in body weight of mice. **(C)** Colon length of mice. **(D)** Assessment of DAI. **(E)** Macroscopic photographs of colons. **(F)** Histopathological score. **(G)** Representative H&E staining pictures of colon tissues (magnification, ×100 ; scale bar = 100 μm and 50 μm). **(H)** The protein expression of Occcludin was detected by Western blotting in colon tissue. **(I)** The protein expression of Occcludin was quantitated by ImageJ software. The data were presented as the means ± SD (n = 8). ^*^ *P* < 0.05, ^**^ *P* < 0.01 and ^***^ *P* < 0.001.

Mice were randomly assigned to five groups: Control, Model, positive drug Mes, XYP-L and XYP-H (n = 8 per group). The Model group was induced with 3% (w/v) DSS in drinking water from day 3–10, while the Control group received plain water. XYP was administered at two dosages (XYP-L and XYP-H) and Mes (600 mg/kg/day) via gavage once daily from day 1 to day 10 (Figure 2A). XYP was dissolved in water, and the final concentration was 0.032 mg/mL and 0.064 mg/mL, respectively. The XYP-L and XYP-H groups were intragastrically administrated with the XYP 0.32 mg/kg and 0.64 mg/kg, respectively, for 2 weeks (Volume = 0.1 mL/10 g according to “Methodology on Chinese medicinal pharmacology” (Wang et al., 2006) and the dosage of traditional Chinese medicine was calculated according to the formula of S_mice_/20 g = 0.0026×S_human_/70kg, the dosage of the high-dose group was twice that of the low-dose group). Mice were monitored daily for weight, stool consistency, and the presence of gross blood in feces or around the anus. Disease Activity Index (DAI) was calculated according to the well-defined criterion (Yao et al., 2025) (Table 2). On day 10, mice were sacrificed by decapitation, and the entire colon was quickly removed.

**TABLE 2:** DAI score.

Score	Weight loss (%)	Stool	Bleeding
0	<1	Normal	Normal
1	1≤∼<5	Softer stool	Weak hemoccult
2	5≤∼<10	Loose stool	Hemoccult
3	10≤∼<15	Moderate diarrhoea	Visual bleeding
4	≥15	Diarrhoea	Gross bleeding

### 2.5 Preparation of colon tissue samples

Following excision, the length of the colon tissue was measured, and the samples were rinsed with 0.9% ice-cold physiological saline. Some samples were fixed in 4% paraformaldehyde (pH 7.0-7.5) for histopathological analysis, while the remaining tissues were stored at −80 °C for future experiments.

### 2.6 Histology and immunohistochemistry

The 4% paraformaldehyde-fixed colon tissues were dehydrated and paraffin-embedded, which were cut into 5 μm sections and stained with hematoxylin and eosin (H&E; G1076, Servicebio, Wuhan, China). After that, the sections were visualized under a pathological section scanner (PerkinElmer, USA). The histological scores were quantified according to (Le et al., 2020). Standard immunohistochemistry (IHC) was performed to assess ACSL4 expression in colon tissue. Primary ACSL4 antibody (1:250, 81196-1-RR, Proteintech) was used. Images were captured in three random fields per sample, with positive staining visualized in brown. Observations were recorded using NIS-Elements 4.0 imaging software (Nikon, Japan), and optical density was quantified with ImageJ (AOD measured across 10 high-power fields; AOD = integrated optical density (IOD) SUM/area SUM).

### 2.7 Transcriptomic analysis

RNA was extracted from mouse colon tissue of Control, Model and XYP-H (as described earlier, n = 3) and subjected to rigorous quality control on the Agilent 2100 Bioanalyzer to detect RNA integrity. Subsequently, a library was built for quality control and preliminary quantification, which were performed using the Qubit2.0 Fluorometer. The insert size of the library was assessed using the Agilent 2100 Bioanalyzer. After the optimal insert size was identified, real-time quantitative polymerase chain reaction (RT-qPCR) was used to quantify the effective concentration of the library to ensure the quality of the library. After inspection, different libraries were pooled according to the effective concentration and the target downstream data volume for Illumina sequencing. Relevant samples were subsequently analyzed for volcano, thermogram, associated GO and KEGG enrichment.

### 2.8 Cell culture

The NCM460 cell line (Cell Bank of Shanghai Institute of Biochemistry & Cell Biology at the Chinese Academy of Sciences) were cultivated in DMEM supplemented with 10% (v/v) FBS, 100 U/ml penicillin and 100 U/ml streptomycin, at 37 °C under a humidified atmosphere with 5% CO_2_. Identification of the cell lines and testing for *mycoplasma* contamination were conducted before their utilization in the experiments.

### 2.9 Preparation of XYP drug-containing serum

Male Sprague-Dawley rats that weighed 220–240 g were purchased from Chengdu Dashuo. All animals were raised in standard cages in a room of constant temperature and humidity (22 °C ± 1 °C; 40%–60%) with a 12:12 dark/light cycle (lights on at 8:00 a.m.; off at 8:00 p.m.). The animals were given free access to food and water throughout the experiment. Rats in the XYP group were intragastrically administered XYP 1.365 g/kg/day for 7 days; meanwhile, rats in the control groups were intragastrically administered equivoluminal saline daily to ensure isocaloric intake. Rats were anesthetized after 1h for the last lavage. Then, blood was taken from the abdominal aorta, centrifuged, in a water bath, sterilized with a filter membrane, and stored in a −80 °C refrigerator, avoiding repeated freezing and thawing.

### 2.10 Cell viability

NCM460 cell viability was measured in 96-well plates. Briefly, 1 × 10^6^ NCM460 cells were incubated in 10% FBS medium per well overnight at 37 °C and 5% CO_2_. The Cell Counting Kit-8 (CCK-8; MeilunBio, Shanghai, China) was used to assess cell cytotoxicity and viability. NCM460 cells were incubated with varying concentrations of XYP drug-containing serum (1%, 2%, 4%, 8%, 16% or 32%) for 24 h. After discarding the supernatant, CCK-8 was added and incubated at 37 °C for 1 h. Absorbance (A) at 450 nm for each treatment group was measured using a microplate reader (Infinite F50, Dicken Trading Co., Ltd., Shanghai, China).

### 2.11 Cell treatment

Based on the results of the cell viability assay, various concentrations of XYP drug-containing serum (4% and 8%) were selected for further investigation. In order to observe the effect of XYP on ferroptosis, NCM460 cells were separated into five groups: control group, model group (RSL3, 15 μM) (Chen et al., 2021), Fer-1 group (Fer-1, 4 μM) (Chen et al., 2021), XYP low-dose group (XYP-L, 4%), and XYP high-dose group (XYP-H, 8%). All treated cells were incubated for 24 h.

### 2.12 Cell transfection

NCM460 cells at around 40%–50% confluence were transfected with Nrf2 siRNA or negative control siRNA using riboFECT™ CP Transfection Kit according to the manufacturer’s instructions. After 24 h of transfection, real-time quantitative RT-qPCR was performed to evaluate the knockdown efficiency. The sequence for siNrf2 is as follows: 5′-GCA​AGA​AGC​CAG​ATA​CAA​A-3′. For the overexpression Nrf2 group, an Nrf2 expression vector (pcDNA3.1-Nrf2) was constructed and confirmed by sequencing. Cell transfections were performed using Lipofectamine 2000 (Invitrogen) according to the manufacturer’s protocol.

### 2.13 Detection of Fe^2+^ release assay and GPX4 enzymatic activity

The total iron content in colon tissues or cells was determined by the ferrous oxazine colorimetric method using an iron assay kit (Shyuanye, Shanghai, China, #R22185) according to the manufacturer’s instructions. The GPX4 enzymatic activity in colon tissues was determined according to the manufacturer’s protocol (MEIMIAN, Jiangsu, China, #MM-44846M2). Spectrophotometric measurements were carried out in 96- well plates.

### 2.14 Detection of oxidative stress (OS) levels

The colon tissues were homogenized in PBS buffer and centrifuged at 3000 rpm for 20 min, and the supernatant was pipetted as the sample to be measured. Then, the reagents were added and incubated according to the instructions of the corresponding kits (MDA, SOD and GSH), and the OD value at the wavelength of 593 nm was detected.

### 2.15 Enzyme-linked immunosorbent assay

The levels of IL-1β, TNF-α and IL-6 in colon tissue were measured using corresponding enzyme-linked immunosorbent assay (ELISA) kits (Multisciences (Lianke) Biotech Co., Ltd., Zhejiang, China) according to the manufacturer’s instructions.

### 2.16 RT-qPCR

Total RNA was extracted from colon tissues or cells using TRIzol reagent (Invitrogen, Carlsbad, CA, USA). Primers (Table 3) were synthesized by Sangon Biotech Co., Ltd. (Shanghai, China). GAPDH was used as the internal reference, and the average mRNA expression in the control group was set to 1. The 2^−ΔΔCT^ method was applied to quantify relative mRNA expression.

**TABLE 3:** Primer sequences of RT-qPCR analyses for mRNA expression.

Gene	NCBI reference	Primer sequences (5′to 3′)	Bp
Nrf2(mouse)	NM_010902.5	F: GCT​CCT​ATG​CGT​GAA​TCC​CAA	143
		R: TTT​GCC​CTA​AGC​TCA​TCT​CGT	
GPX4 (mouse)	NM_008162.4	F: CAT​CGA​CGG​GCA​CAT​GGT​CT	138
		R: CCA​CAC​TCA​GCA​TAT​CGG​GCA​T	
FTH1(mouse)	NM_010239.2	F: CCA​TCA​ACC​GCC​AGA​TCA​AC	85
		R: GAA​ACA​TCA​TCT​CGG​TCA​AA	
SLC7A11 (mouse)	NM_011990.2	F: CCT​GGC​ATT​TGG​ACG​CTA​CA	378
		R: GCA​AGG​GGG​ATG​GTT​TTT​TC	
ACSL4 (mouse)	NM_019477.3	F: TTA​CCT​ATG​GCT​GTA​GGA​TTG​GAT	98
		R: CAG​TAC​AGT​ACA​ATC​ACC​CTT​GCT	
Nrf2(human)	NM_001313902.2	F: CAA​CTC​AGC​ACC​TTA​TAT​CTC​G	116
		R: ACA​AGG​AAA​ACA​TTG​CCA​TC	
GPX4 (human)	XM_057300686.2	F: CGC​CTT​TGC​CGC​CTA​CTG​AAG​C	150
		R:AACCATGTGCCCGTCGATGTCC	

### 2.17 Western blotting analysis

Total protein from colon tissues/NCM460 cells was extracted using RIPA buffer containing a protease and phosphatase inhibitor cocktail. The Western blotting procedure followed the same steps as previously described. ImageJ software was used for analysis and quantification. β-actin served as an internal control for total protein normalization. All antibodies and their details used in Western blotting analysis are presented in Table 3.

### 2.18 Flow cytometry analysis

For flow cytometry analysis, NCM460 cells were treated as indicated. Cells were harvested, twice washed in PBS, and stained by the annexin V-FITC/propidium iodide (PI) apoptosis assay kit (Bioscience, Shanghai, China) following the manufacturer’s instructions. A BD FACSAria III flow cytometer was used to analyze stained cells, and FlowJo software was used to process the data.

### 2.19 Immunofluorescence (IF) staining

All groups of cells on glass coverslips were fixed with 4% paraformaldehyde for 20 min and washed with 0.01 M PBS. Cells were permeabilized with 0.3% Triton X-100 in PBS, blocked with 3% BSA in PBS for 2 h, and then incubated with an Nrf2 primary antibody (1:5 000, ab62352, Abcam) overnight at 4 °C. After washing with PBS, cells were incubated with a secondary antibody for 2 h at 37 °C. 4′,6-Diamidino- 2-phenylindole (DAPI; C1002, Beyotime, Shanghai, China) was employed to stain the nuclei. The fluorescence images were taken using an inverted fluorescence microscope (Olympus BX53, Japan). Image-Pro Plus software was used to quantify the protein in a blinded manner, measuring the average and the area of fluorescence intensity.

### 2.20 Determination of intracellular ROS levels

NCM460 cells were incubated with H_2_DCFDA as described in the ROS analysis kit. Then, the intracellular ROS levels were analyzed by flow cytometer and expressed as mean fluorescence intensity (Beckman, USA).

### 2.21 Statistical analysis

All statistical analyses were performed using GraphPad Prism (San Diego, CA, USA) and SPSS 21.0 (IBM, NY, USA). Data are presented as mean ± standard deviation (SD). Tukey’s *post hoc* test and one-way analysis of variance (ANOVA) were applied to determine differences in biochemical parameters between groups. A p-value of less than 0.05 was considered statistically significant.

## 3 Results

### 3.1 XYP alleviated DSS-induced UC in mouse model

In this study, the DSS-induced UC mouse model was successfully established and applied to explore the protective effect of XYP on UC. During this experiment, typical pathological features of UC were observed in DSS-induced mice, including reduced diet, weak movements, diarrhea, and blood in stool. Additionally, compared to the control mice, UC mice showed decreased body weight, increased DAI scores, and shortened colon length (Figures 2B–E). H&E staining showed severe damage in the colon tissues of UC mice, such as mucosal ulceration, crypt destruction, and inflammatory cell infiltration (red arrow). However, these pathological characteristics were markedly alleviated by highdose XYP (Figures 2F,G). The low expression of tight junction (TJ) proteins, Occludin is critical in triggering intestinal barrier damage, which promotes inflammatory outbreaks (Schlegel et al., 2021). Consequently, the influence of XYP on the expression of TJ proteins in UC mice was further explored. According to the results, the protein level of occluding was notably decreased in UC mice compared to the control mice, which were remarkably reversed by XYP (Figures 2H,I). These data suggested that XYP could alleviate UC in mice.

### 3.2 Inhibition of XYP on pro-inflammatory cytokines in UC mice

**FIGURE 3:**
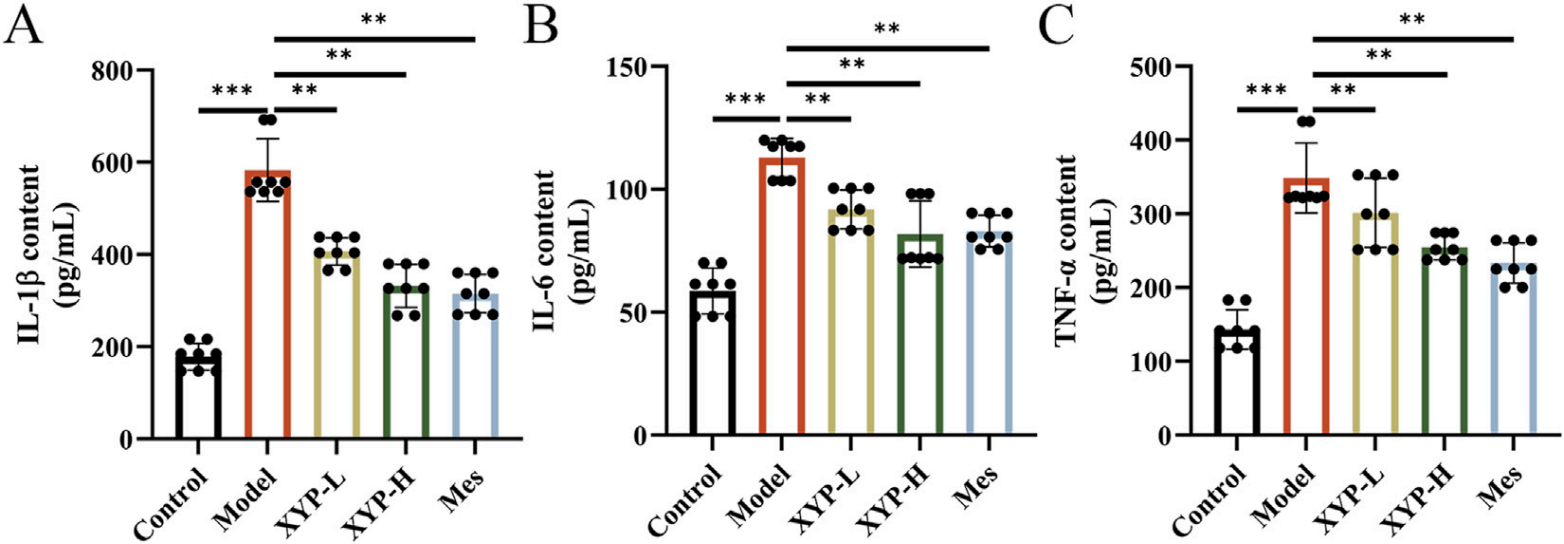
Effects of XYP on intestinal inflammation in UC mice. **(A–C**) The levels of IL-1β, IL-6, and TNF-α in colon tissues. The data were presented as the means ± SD (n = 8). ^*^ *P* < 0.05, ^**^ *P* < 0.01 and ^***^ *P* < 0.001.

Subsequently, the contents of pro-inflammatory cytokines in colon tissues were measured to evaluate the mitigation of XYP on the inflammation in UC mice. Consistently, the levels of IL-1β, IL-6, and TNF-α in UC mice were remarkably higher than those in control mice. However, the levels of these cytokines were notably downregulated by XYP (Figures 3A–C).

### 3.3 Transcriptomics analysis of XYP-treated UC mice

**FIGURE 4:**
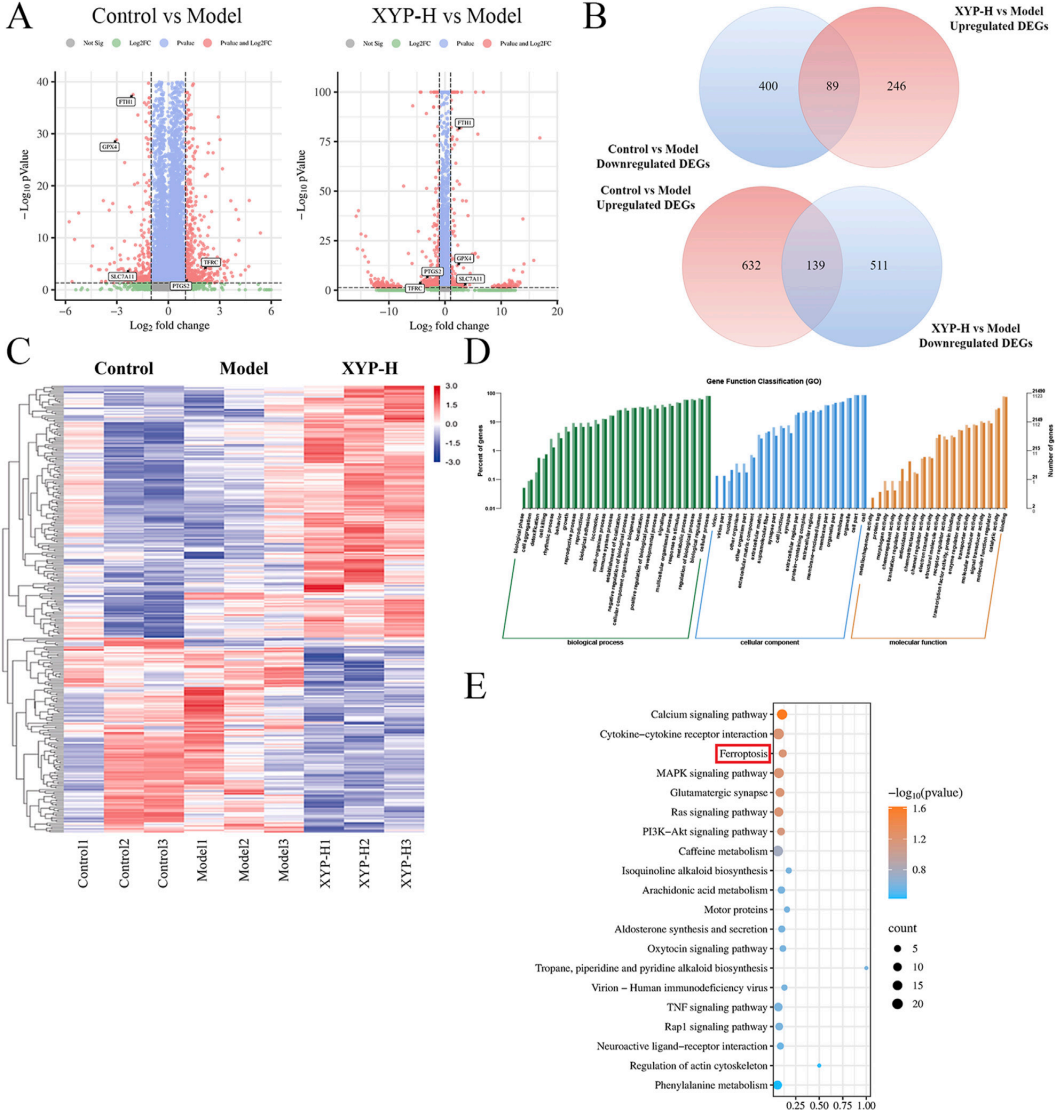
Transcriptome analysis reveals the signaling pathways involved in the therapeutic effect of XYP on UC mice. **(A)** The volcano map displayed the number of DEGs when comparing the model group to the control group and the XYP-H group to the model group. **(B)** Venn diagrams displayed the number of DEGs that were upregulated in model group (Model vs. Control, Upregulated DEGs) and downregulated by XYP (XYP-H vs. Model, Downregulated DEGs), along with DEGs that were downregulated in model group (Model vs. Control, Downregulated DEGs) and upregulated by XYP (XYP-H vs. Model, Upregulated DEGs). **(C)** Heatmap showing the DEGs in the model group that were reversed by XYP treatment. **(D)** GO enrichment analysis of the DEGs in the model group that were reversed by XYP treatment. **(E)** The top 20 signaling pathways in KEGG enrichment analysis of the DEGs in the model group that were reversed by XYP treatment. The number of animals: transcriptomics analysis (n = 3 per group).

A total of 21,452 expressed genes were detected. Compared to the control mice, the UC mice exhibited 461 DEGs, with 142 DEGs being upregulated and 319 DEGs being downregulated. When comparing the XYP-treated mice to the UC mice, there were 95 DEGs identified, with 63 DEGs being upregulated and 32 DEGs being downregulated (Figure 4A). Venn diagrams were constructed to visualize the intersection between these two sets of DEGs, revealing a total of 89 and 139 overlapping genes, respectively (Figure 4B). The heatmap results indicated that all groups were well separated from each other, and the XYP-H group showed a trend of reverting to the control group (Figure 4C). Then 95 DEGs (XYP-treated vs. UC) were then subjected to GO and KEGG pathway enrichment analyses. The GO enrichment analysis revealed that the intervention of XYP influenced the production and degradation of the extracellular matrix, as well as the metabolism of iron ions (Figure 4D). Similarly, the KEGG pathway analysis indicated significant enrichment of ferroptosis (Figure 4E). These findings suggest that XYP may alleviate UC by regulating ferroptosis-related signaling pathways.

### 3.4 XYP ameliorated colonic ferroptosis and OS in UC mice

**FIGURE 5:**
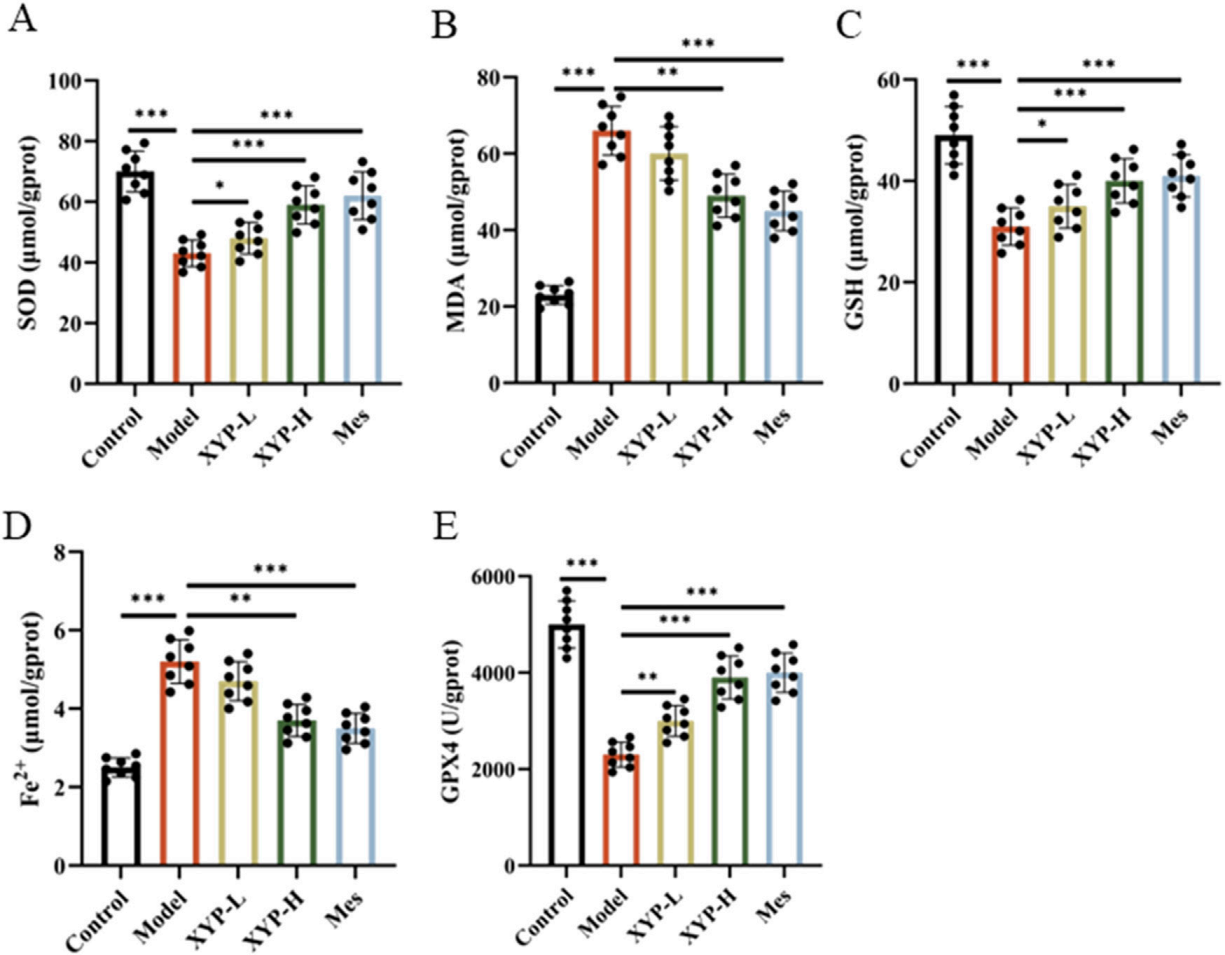
Inhibition of XYP on the ferroptosis and OS in UC mice. **(A–C)** The level of SOD, MDA and GSH in colon tissues; **(D)** The level of iron in colon tissues; **(E)** The level of GPX4 enzymatic activity in colon tissues. The data were presented as the means ± SD (n = 8). *P < 0.05, **P < 0.01 and ***P < 0.001.

Based on the findings of transcriptomics results, whether the therapeutic effect of XYP on colitis is also involved in the pathway of ferroptosis and oxidative stress, we examined the levels of Fe, GPX4, GSH, SOD and MDA. Our findings revealed a significant decrease in GPX4, SOD and GSH levels, accompanied by a significant increase in Fe^2+^ and MDA levels, in the DSS group when compared to the control group (Figures 5A–E). However, following the administration of XYP, the aforementioned indexes were restored to a level approaching that of the control group.

### 3.5 XYP attenuated ferroptosis by modulating the Nrf2/GPX4 pathway *in vivo*

**FIGURE 6:**
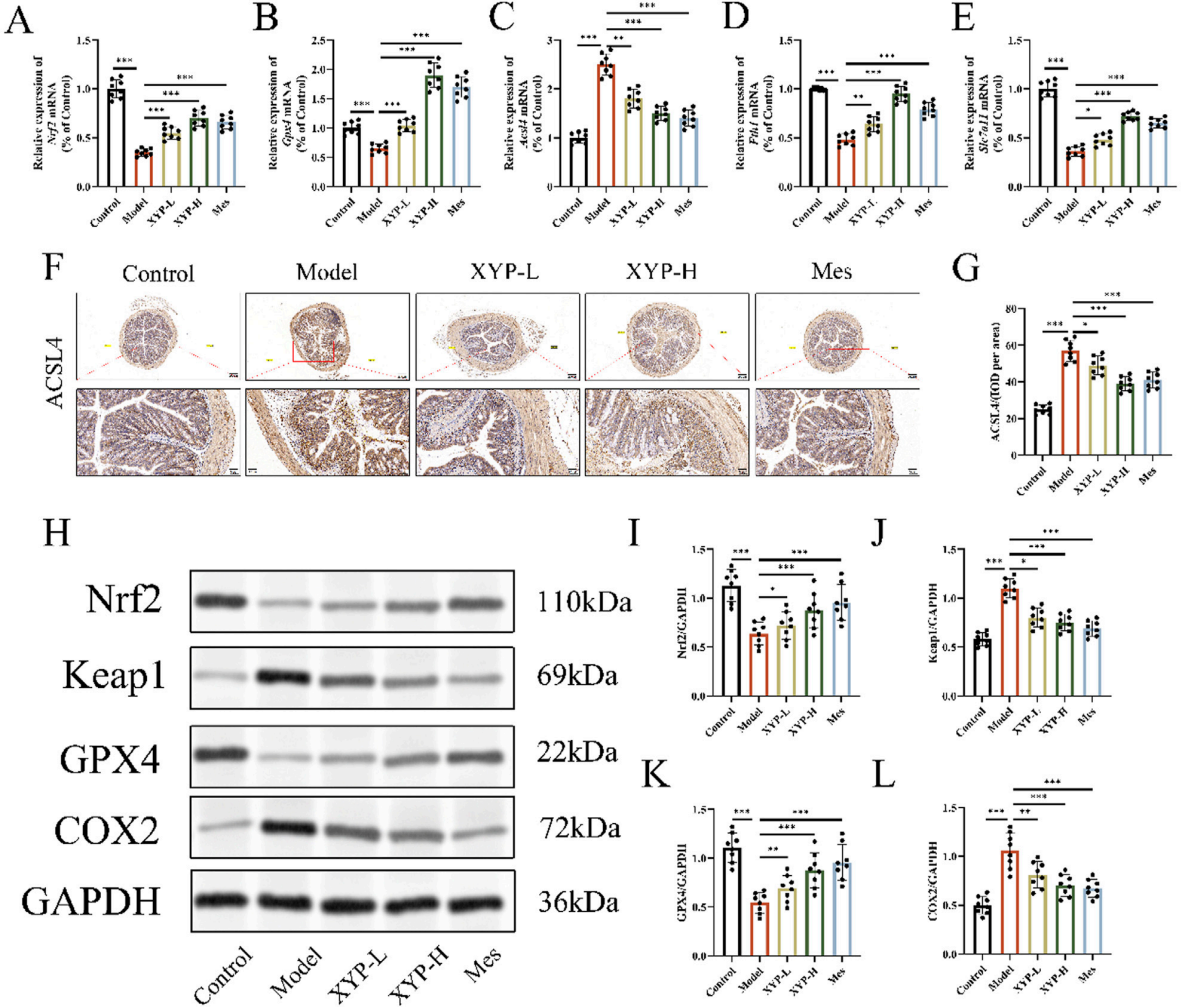
XYP attenuated ferroptosis by modulating the Nrf2/GPX4 pathway *in vivo*. **(A–E)** The mRNA expression of *Nrf2*,*Gpx4, Acsl4, Fth1* and *Slc7all* in colon tissues. **(F)** Protein expression of ACSL4 quantified using AOD by Image-Pro Plus 6.0 software. Similar results were observed in three independent experiments. **(G)** Protein expression of ACSL4 in the colon tissue of mice in each group using IHC staining (magnification, ×100 ; scale bar = 200 μm and 50 μm). **(H)** The protein expression of Keap1, Nrf2, GPX4 and COX2 were detected by Western blotting in colon tissue. **(I–L)** The protein expression of Keap1, Nrf2, GPX4 and COX2 were quantitated by ImageJ software. The data were presented as the means ± SD (n = 8). ^*^ *P* < 0.05, ^**^ *P* < 0.01 and ^***^ *P* < 0.001.

To further substantiate our hypothesis that XYP mitigates UC in mice by regulating ferroptosis-related signaling pathways, we conducted an assessment of key ferroptosis markers within colonic tissue using IHC, Western blotting and RT-qPCR. The RT-qPCR data revealed that, relative to the control group, there was a significant decrease in the mRNA expression of *Nrf2*, *Gpx4*, *Fth1* and *Slc7a11*, as well as a great increase in the mRNA expression of *Acsl4* in the colonic tissue of UC model mice. Comparatively, the XYP-treated groups exhibited a pronounced increase in the mRNA levels of *Nrf2*, *Gpx4*, *Fth1* and *Slc7a11* and a significant decrease in the mRNA expression of *Acsl4* (Figures 6A–E). Additionally, the IHC results highlighted weak positive signals for Nrf2 and GPX4 in the colonic tissues of UC model mice, underscoring the involvement of the Nrf2/GPX4 pathway in the development of UC. Notably, treatment with XYP significantly increased the expression of Nrf2 and GPX4 in comparison to the model group (Figures 6F,G). Consistent with these findings, Western blotting analyses demonstrated that XYP treatment led to a significant increase in the protein levels of Nrf2 and GPX4, as well as a great decrease in the protein levels of Keap1 and COX2 (Figures 6H–L). These outcomes indicate that XYP alleviates inflammation in UC mice by modulating ferroptosis through its regulation of Nrf2/GPX4 pathway.

### 3.6 Protective effect of XYP on NCM460 cells induced by RSL3

**FIGURE 7:**
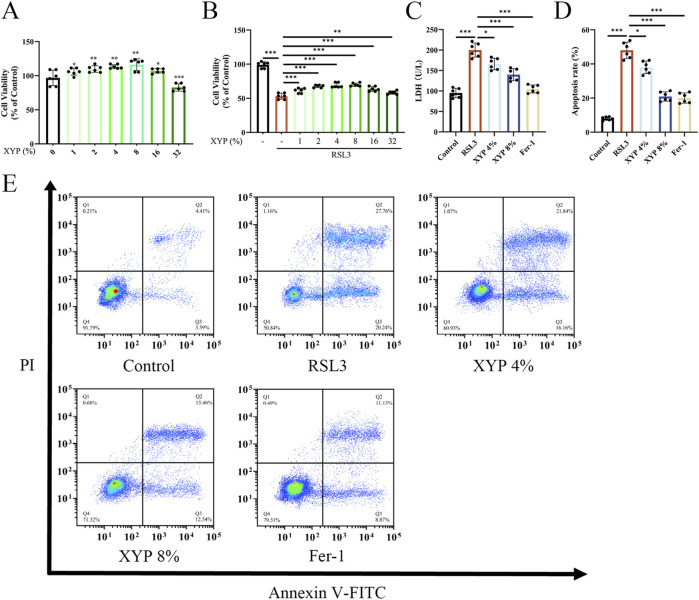
Protective effect of XYP on NCM460 cells induced by RSL3. **(A)** Cytotoxicity of various concentrations of XYP drug-containing serum on NCM460 cells in 24 h. **(B)** Cell viability of various concentrations of XYP drug-containing serum on NCM460 cells treated with or without RSL3 in 24 h. **(C)** Content of LDH in the supernatant of NCM460 cells. **(D)** The apoptosis rate of cells in each group. **(E)** Flow cytometry analysis of Annexin V-FITC/PI staining for the determination of apoptosis in NCM460 cells. The data were presented as the means ± SD (n = 6). ^*^ *P* < 0.05, ^**^ *P* < 0.01 and ^***^ *P* < 0.001.

To gain further insight into the inhibitory effect of XYP on ferroptosis, an examination was conducted at the cellular level to determine whether XYP attenuated RSL3-induced ferroptosis in NCM460 cells (Yuan et al., 2025; Liang et al., 2025). RSL3 was observed to significantly alter the growth and morphology of NCM460 cells. Following the administration of XYP drug-containing serum at varying concentrations to NCM460 cells for a period of 24 h, no discernible impact on cellular activity was observed until the concentrations exceeded 32%. Then, the NCM460 cells were treated with RSL3 to establish the ferroptosis model to explore the role of XYP on ferroptosis *in vitro*. The results showed that the cell activity inhibited by RSL3 was significantly reversed with increasing concentrations of XYP (Figures 7A,B). Compared to the RSL3 group, the 8% XYP drug-containing serum treatment group achieved the highest survival rate. Based on these results, concentrations of 4% and 8% were chosen as the low and high doses for subsequent *in vitro* experiments. Moreover, the LDH content in supernatants were remarkably decreased by XYP (Figure 7C). Consistently, the apoptosis rate of RSL3-induced NCM460 cells were strikingly higher than those in the control cells, which were significantly suppressed by XYP treatment (Figures 7D,E). The same trends were also observed in Fer-1 treated cells. All these findings suggest that XYP provides effective protection against RSL3-induced cellular injury.

### 3.7 XYP attenuated ferroptosis in RSL3-induce NCM460 cell via activating Nrf2/Gpx4 signaling pathway

**FIGURE 8:**
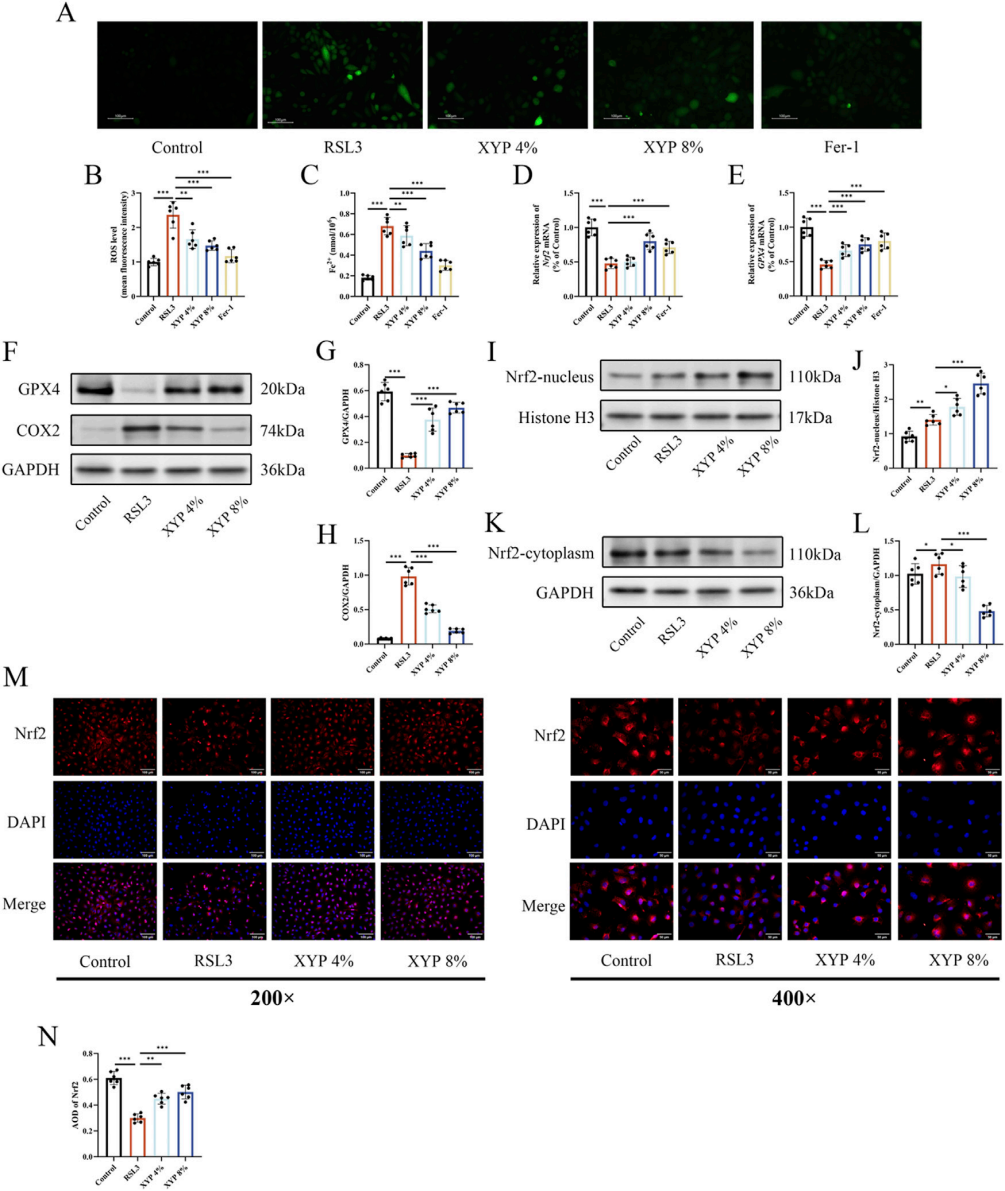
XYP attenuated ferroptosis in RSL3-induce NCM460 cell via activating Nrf2/Gpx4 signaling pathway. **(A)** Representative images of DCFH-labeled fluorescence microscopy. Scale bar = 100 μm. **(B)** The level of ROS in NCM460 cells. **(C)** The level of iron in each group of cells. **(D)** The mRNA expression of *Nrf2* in each group of cells. **(E)** The mRNA expression of *GPX4* in each group of cells. **(F)** The protein expression of GPX4 and COX2 in each group of cells. **(G,H)** The protein expression of GPX4 and COX2 was quantitated by ImageJ software. **(I)** The protein expression of nucleus-Nrf2 in each group of cells. **(J)** The protein expression of nucleus-Nrf2 was quantitated by ImageJ software. **(K)** The protein expression of cytoplasm-Nrf2 in each group of cells. **(L)** The protein expression of cytoplasm-Nrf2 was quantitated by ImageJ software. **(M)** Immunofluorescence staining of Nrf2 (200× and 400×). Merged images of DAPI for the nucleus (blue) and Nrf2 immunofluorescence (red). Similar results were observed in three independent experiments. Scale bar = 100 and 50 μm. **(N)** Quantification of the relative fluorescence intensities of Nrf2 using AOD by Image-Pro Plus 6.0 software. The data were presented as the means ± SD (n = 6). ^*^ *P* < 0.05, ^**^ *P* < 0.01 and ^***^ *P* < 0.001.

To assess ROS accumulation, H_2_DCFDA probes were used in this study. Microscopic observations demonstrated a significant increase in fluorescence intensity following RSL3 treatment, indicating ROS accumulation in an inflammatory environment. On the other hand, XYP treatment dose-dependently reduced ROS accumulation (Figures 8A,B). The Fe^2+^ level in RSL3-induce NCM460 cells was remarkedly elevated. After treatment with XYP, the Fe^2+^ level was greatly decreased (Figure 8C). To further confirm whether the attenuation of XYP on ferroptosis is via activating Nrf2/Gpx4 signaling pathway *in vitro*, we evaluated the expression of induces associated to ferroptosis. RSL3 treatment decreased the mRNA levels of *Nrf2* and *Gpx4* (Figures 8D,E), while XYP treatment effectively attenuated this downregulation. Moreover, the protein expression of GPX4 were significantly decreased after stimulation with 15 μM RSL3, while the protein expression of COX2 were significantly increased. After treatment with XYP, the levels of above proteins were significantly reversed (Figures 8F–H). Further exploration of the effect of XYP on nuclear translocation of Nrf2 was conducted. As illustrated in Figures 7I–L, similar to other studies (Fang et al., 2019; Wang et al., 2024), the protein expression of Nrf2 was markedly increased in the nucleus and decreased in the cytosol following exposure to RSL3. Interestingly, treatment with XYP further enhanced the expression of Nrf2 in the nucleus and significantly reduced its expression in the cytoplasm. Furthermore, the IF results supported the aforementioned findings, indicating a notable decline in Nrf2 expression in RSL3-stimulated cells, which was accompanied by a marked elevation following XYP treatment (Figures 8M,N). These results suggest that XYP attenuated ferroptosis in RSL3-induce NCM460 cell via activating Nrf2/Gpx4 signaling pathway.

### 3.8 Nrf2 served as the key target in the effect of XYP on attenuating ferroptosis

**FIGURE 9:**
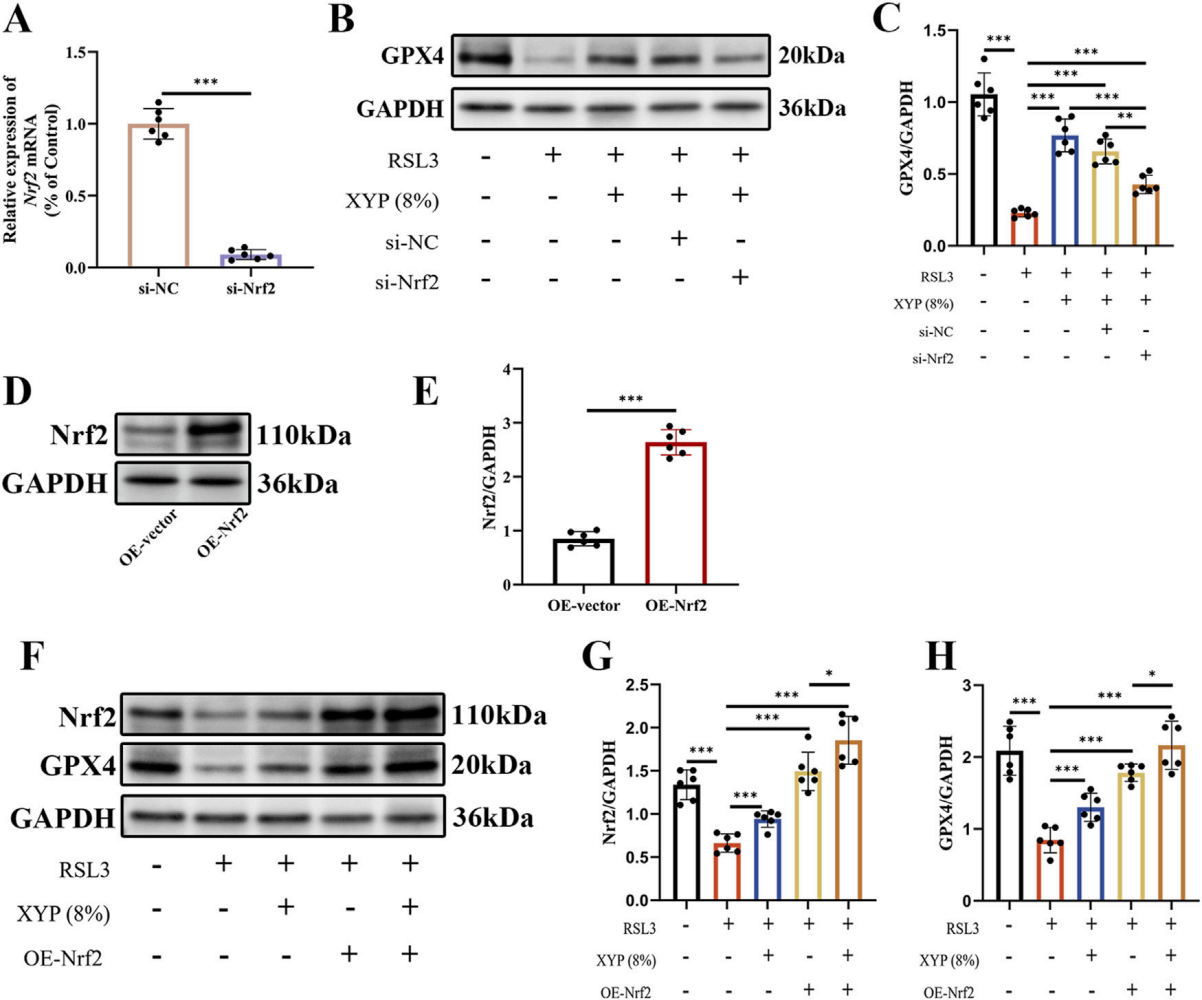
Nrf2 served as the key target in the effect of XYP on attenuating ferroptosis. **(A)** Validation of si-Nrf2 silencing effectiveness through RT-qPCR. **(B)** The protein expression of GPX4 was detected by Western blotting in each group of cells. **(C)** The protein expression of GPX4 was quantitated by ImageJ software. **(D)** Validation of Nrf2 overexpression effectiveness through Western blotting. **(E)** The protein expression of Nrf2 was quantitated by ImageJ software. **(F)** The protein expression of Nrf2 and GPX4 was detected by Western blotting in each group of cells. **(G–H)** The protein expression of Nrf2 and GPX4 were quantitated by ImageJ software. The data were presented as the means ± SD (n = 6). ^*^ *P* < 0.05, ^**^ *P* < 0.01 and ^***^ *P* < 0.001.

Nrf2 is an oxidative stress-induced transcription factor that negatively regulates ferroptosis (Dodson et al., 2019). Nrf2/GPX4 is considered a crucial signaling pathway for the regulation of ferroptosis. The effectiveness of the specific si-Nrf2 sequence employed in this study was confirmed through RT-qPCR, which revealed a great reduction in Nrf2 expression in NCM460 cells (Figure 9A). As shown in Figures 9B,C, Western blotting analysis revealed that RSL3 downregulated GPX4 protein expression in NCM460 cells, which was effectively reversed by XYP. Notably, this effect was partially abolished upon Nrf2 silencing. Additionally, Nrf2 was overexpressed to strengthen the hypothesis by using pcDNA3.1-Nrf2. The effectiveness of the specific OE-Nrf2 plasmid employed in this study was confirmed through Western blotting, which revealed a great increase in Nrf2 expression in NCM460 cells (Figures 9D,E). As shown in Figures 9F–H, Western blotting analysis revealed that RSL3 downregulated Nrf2 and GPX4 protein expression in NCM460 cells, which were effectively reversed by XYP. Notably, this effect was also detected in OE-Nrf2 NCM460 cells. Overexpression of Nrf2 resulted in a significantly greater increase in the protein expression of Nrf2 and GPX4 in RSL3-exposed NCM460 cells, while XYP further enhanced such effect. These results indicated that Nrf2 served as the key target in the effect of XYP on attenuating ferroptosis.

## 4 Discussion

UC has become an inflammatory disease that has attracted widespread public attention in recent years (Ordás et al., 2012). Its causative factors are complex and diverse, and its long-term development can lead to colorectal cancer, which has become the third most common cancer in the world, accounting for 10% of cancer deaths (Dekker et al., 2019). Therefore, prevention and early treatment of ulcerative colitis is of great importance. Nevertheless, the current range of therapeutic options for UC is limited, necessitating the pursuit of novel, safe and effective therapeutic interventions.

Clinically, XYP can improve symptoms of UC and enhance the quality of life in patients (Liu et al., 2021). Currently, the DSS-induced UC mouse model is extensively applied, mainly for its easily controllable and reproducible establishment conditions and the similarity of symptoms in this model to those of UC patients (Low et al., 2013). Therefore, DSS-induced UC mice were used to evaluate the therapeutic effects of XYP. Generally, UC is characterized by severe intestinal histological changes, inflammation, and barrier disruption (Le et al., 2020). IL-6, IL-1β, and TNF-α are classical pro-inflammatory cytokines that contribute to the development of intestinal epithelial injury (Yin et al., 2021). TJ proteins Occludin is the principal structural components of the intestinal barrier, and their aberrant expression can indicate intestinal barrier damage (Schlegel et al., 2021). It is well known that large amounts of pro-inflammatory cytokines can disturb the integrity of the intestinal barrier and further exacerbate inflammation (Abraham et al., 2022). In this study, XYP could markedly inhibit colonic pathological injury, decrease inflammatory cytokines levels, and increase the protein expression of Occludin in UC mice, demonstrating that XYP could attenuate UC.

XYP as a widely recognized TCM formula, contains a diverse array of compounds (Wang et al., 2006). To elucidate the material basis for its anti-UC effects and its unique mode of action, it is essential to understand its mechanisms of action. We further adopted a multi-omics approach to predict potential mechanisms of XYP against UC. Through transcriptomics analysis, we found that the differential genes after XYP intervention were significantly enriched in ferroptosis. After performing enrichment analysis on the DEGs, we again found that ferroptosis were significantly enriched. These findings strongly suggest that XYP may improve UC by regulating the process of ferroptosis.

The present study demonstrated that UC was closely related to ferroptosis by comprehensively analyzing ferroptosis-related genes in transcriptomic data from human colon samples (Rochette et al., 2022). Ferroptotic cells can also promote inflammatory responses by releasing damage-associated molecular patterns, and the process is mainly evoked with iron and lipid peroxidation products. Iron is in dynamic homeostasis under physiological conditions, while overloaded iron can cause pathological damage (Jiang et al., 2021). For instance, excessive iron in the gut was demonstrated to be susceptible to intestinal inflammation and microbial disorder, thereby exacerbating UC, which could be mitigated by iron chelators (Mahalhal et al., 2021; Minaiyan et al., 2012). Additionally, iron can catalyze the oxidation of polyunsaturated fatty acids by increasing the activity of arachidonate lipoxygenase, which ultimately produces a variety of toxic aldehydes, such as MDA and GSH (Li et al., 2020). MDA, the most abundant lipid peroxidation product, is commonly used as a biomarker to appraise the degree of lipid peroxidation (Toto et al., 2022). Notably, MDA can function as an inflammatory mediator to elicit abnormal intestinal immune responses in UC (Zhang X. et al., 2023). Overall, iron and MDA are critical indicators for ferroptosis and are also strongly correlated with the severity of UC. Increasing evidence has indicated that ferroptosis is primarily driven by the inactivation of the antioxidant system, involving OS (Galaris et al., 2019). Usually, the degree of OS is evaluated by the GSH content and SOD activity (Demirci-Çekiç et al., 2022). GSH, a most abundant intracellular antioxidant, can be oxidized to GSSG by reducing reactive oxygen species (ROS) (Golubkova et al., 2023). SOD, an enzymatic antioxidant, catalyzes the transformation of superoxide to hydrogen peroxide and oxygen, thereby reducing ROS production (Henning et al., 2022). Particularly, the deficiency of antioxidant molecules is closely correlated with the progression of UC (Tan et al., 2022). It has been confirmed that GSH synthesis is deficient in the intestines of UC patients (Tan et al., 2022). Therefore, ferroptosis in UC can be inhibited by enhancing the antioxidant capacity of enterocytes.

*In vivo*, we confirmed the inhibitory effect of XYP on ferroptosis through detection of key markers of ferroptosis. We found that the Fe^2+^ level in the colon tissues of UC mice was significantly increased, while the GPX4 enzymatic activity in colon tissues was greatly reduced. XYP treatment significantly improved these conditions. Meanwhile, the level of oxidative stress marker MDA was significantly elevated in the colon tissues of UC mice, while the level of SOD and GSH were decreased. Treatment with limonin reduced the levels of MDA, restored the GSH and SOD content, and enhanced antioxidant capabilities. These findings are consistent with the excellent antioxidant effects of XYP reported in early studies (Li et al., 2022; Chen et al., 2020). In addition, the intestinal barrier plays a pivotal role in maintaining the stability of the internal environment of the intestinal mucosa. It is primarily composed of NCM460. Whereas previous studies have shown that iron overload induces NCM460 dysfunction, leading to structural damage and inflammatory disease progression (Yuan et al., 2025). So, for establishing an *in vitro* model, RSL3 was employed to induce ferroptosis in NCM460 cells, which were then treated with varying concentrations of XYP. Importantly, this effect was comparable to that observed with the positive control substances, the ferroptosis inhibitor Fer-1. The results indicated that XYP could provide effective protection against RSL3-induced cellular injury by reducing apoptosis. All of the aforementioned indicating that XYP is capable of slowing the occurrence of ferroptosis.

GPX4 plays a pivotal role in the regulation of ferroptosis, facilitating the detoxification of lipid peroxidation and exerting an anti-ferroptosis effect. Nrf2 is a key transcriptional target of GPX4, which directly or indirectly affects the antioxidant function of GPX4 (Liu et al., 2023). Under normal conditions, Nrf2 is bound to its repressor Kelch-like epichlorohydrin-associated protein 1 (Keap1) in the cytoplasm, and the Keap1-Nrf2 protein complex is inactivated. Upon OS, Nrf2 dissociates from Keap1, translocated to the nucleus, and activates the expression of downstream antioxidant response element-containing genes (Dong et al., 2020). To date, studies have identified an association between UC and the Nrf2/GPX4 signaling pathway. Furthermore, reports have demonstrated that the Nrf2/GPX4 pathway is activated in UC (Bian et al., 2024; Chen et al., 2024). It has also been reported that metformin can promote the activation and nuclear translocation of Nrf2 in lipopolysaccharide (LPS)-induced colitis, thereby increasing the expression of antioxidants (HO-1) and suppressing oxidative stress (Wu et al., 2018). Moreover, erastin, a ferroptosis agonist, induced ferroptosis in a lung cancer cell model by inhibiting Nrf2 (Kwon et al., 2020). However, these chemical Nrf2 activators tend to directly and often aggressively activate Nrf2, which lead to pro-tumorigenic effects in some contexts (Houghton et al., 2016). Furthermore, chemical Nrf2 activator could only address the ferroptosis aspect of UC. Considering the XYP is consist of a complex mixture of bioactive compounds, which may create a gentle, sustained, and potentially more physiological activation compared to the potent, sometimes off-target, effects of synthetic agonists. It is more meaningful to explore whether the therapeutic effect of XYP on attenuating ferroptosis was via activating Nrf2/GPX4 signaling pathway. To elucidate the exact molecular mechanism of XYP’s action, we conducted RT-qPCR, Western blotting, IHC or IF experiments to verify it. *In vivo*, RT-qPCR, IHC and Western blotting analyses revealed that the mRNA and protein expression of GPX4 and Nrf2 were markedly suppressed in UC mice, while Keap1 and COX2 was markedly increased. Conversely, the disrupted expression was restored by the intervention of XYP. Consistently, *in vitro*, the mRNA expression of GPX4 and Nrf2 were also upregulated by XYP. Moreover, treatment with XYP partially restored Nrf2 expression and nuclear accumulation. To further elucidate the role of Nrf2 in the effect of XYP on attenuating ferroptosis, Nrf2 was silenced in NCM460 cells by siRNA. Notably, in the absence of Nrf2, XYP was unable to inhibit ferroptosis in NCM460 cells, which underscores the critical role of Nrf2 activation in mediating the pharmacological effects of XYP. However, the overexpression of Nrf2 in NCM460 cells could also reversed the effect induced by RSL3 by activating Nrf2/GPX4 pathway. The presented evidence substantiates the assertion that XYP can activate the Nrf2/GPX4 signaling pathway, thereby inhibiting ferroptosis, while Nrf2 served as the key target in the process.

This study provides new insights into the mechanism of action of XYP in the treatment of UC, enriching the theoretical framework of the mechanisms of TCM formulations. It emphasizes the potential of XYP as a multi-target therapeutic strategy that not only improves intestinal function but also effectively relieves inflammation and oxidative stress. More importantly, the novelty of our study is the causal link: XYP reduces inflammation → which in turn reduces the cellular trigger for ferroptosis → leading to a more resilient colon epithelium. While synthetic Nrf2 agonists act with high affinity on a single target, our data suggest that XYP’s action is more nuanced. We propose that its effect is not merely one of potent Nrf2 activation, but of “system modulation”. Beyond upregulating Nrf2 and GPX4, our data show that XYP also significantly modulated key inflammatory cytokines like TNF-α and IL-6 that are known ferroptosis inducers. This multi-target engagement suggests XYP simultaneously bolsters the cellular antioxidant defense at multiple points, potentially leading to a more robust and resilient anti-ferroptotic effect than single-agent approaches. Besides, current UC treatments often focus on suppressing immune hyperactivity. Our findings contribute a new dimension to this understanding by illustrating that protecting the colon epithelium from a specific, inflammation-triggered cell death (ferroptosis) is a viable therapeutic strategy. More importantly, XYP embodies a ‘host-centric’ therapeutic strategy. By strengthening the intestinal barrier against ferroptosis, it may not only reduce cell death but also subsequently decrease the exposure of the immune system to luminal antigens, thereby breaking a vicious cycle of inflammation. This positions XYP not just as an anti-ferroptotic agent, but as a system stabilizer that aligns with the holistic principles of TCM. All these provides new directions for the clinical management of patients with UC. Particularly in the exploration of TCM treatments, this may guide more targeted research and clinical trials.

## 5 Conclusion

Overall, our study confirmed XYP prominently alleviated DSS-induced colon injury with improving colon pathological damage, relieving inflammation and oxidative stress. Moreover, XYP alleviated ferroptosis in colon injury by activating the Nrf2 signaling pathway. These findings provide new insight and experimental evidence for the treatment of UC with TCM formula.

## Data Availability

The original contributions presented in the study are publicly available. This data can be found here: NCBI BioProject, accession number PRJNA1371368 (https://www.ncbi.nlm.nih.gov/bioproject/PRJNA1371368).
